# Cytomegalovirus Enteritis after Surgery for Acute Type A Aortic Dissection Complicated by Malperfusion and Thymoma: A Case Report

**DOI:** 10.70352/scrj.cr.26-0318

**Published:** 2026-10-08

**Authors:** Hana Fukuta, Masahiko Narita, Ryohei Ushioda, Tomoki Nakatsu, Takayuki Kadohama, Hiroyuki Kamiya

**Affiliations:** 1 Department of Cardiovascular Surgery, Seitetsu Memorial Muroran Hospital, Muroran, Hokkaido, Japan; 2 Department of Cardiac Surgery, Asahikawa Medical University, Asahikawa, Hokkaido, Japan

**Keywords:** cytomegalovirus, enteritis, acute aortic dissection, malperfusion, thymoma, immunosuppression

## Abstract

**INTRODUCTION:**

Cytomegalovirus (CMV) enteritis is a potentially life-threatening gastrointestinal infection typically observed in immunocompromised individuals, such as transplant recipients or patients receiving chemotherapy or long-term corticosteroids. However, CMV enteritis can also develop in the absence of overt immunosuppression, particularly in elderly or critically ill patients under physiological stress, nutritional decline, or prolonged hospitalization. Diagnosis in these settings is challenging because clinical symptoms and imaging findings are nonspecific and may overlap with ischemic or other infectious enteritis.

**CASE PRESENTATION:**

We report a rare case of CMV enteritis in a 65-year-old man who underwent emergent surgical repair for acute type A aortic dissection complicated by superior mesenteric artery (SMA) malperfusion. He underwent total arch replacement, aortic valve replacement with a bioprosthetic valve, and concomitant thymectomy for an anterior mediastinal mass. On POD 4, additional endovascular interventions were performed, including thoracic endovascular aortic repair and SMA stenting. His postoperative course was notable for persistent ileus, poor oral intake, progressive nutritional deterioration, and sustained postoperative fever with elevated inflammatory markers. Because these symptoms and inflammatory findings persisted without an identified infectious source, CMV testing was performed on POD 20. The results, which became available on POD 23, showed positive CMV antigenemia (C7-HRP: 42/50000 WBC) and CMV DNA (1.2 × 10^4^ IU/mL). Abdominal CT demonstrated diffuse small-bowel wall thickening and submucosal edema. Because of his clinical status, endoscopic biopsy was deferred, and CMV enteritis was diagnosed presumptively based on clinical, radiologic, and virologic findings. Intravenous ganciclovir (300 mg every 12 hours) was initiated and continued for 22 days, resulting in gradual improvement in abdominal symptoms, a marked decrease in CMV DNA levels, and conversion of CMV antigenemia to negative. He was transferred to another facility on POD 90.

**CONCLUSIONS:**

CMV enteritis should be considered as a cause of unexplained postoperative fever after open-heart surgery, particularly following repair of acute aortic dissection complicated by malperfusion-related intestinal ischemia, even without classic immunosuppressive conditions. Surgical stress, ischemic bowel injury, and delayed nutritional recovery may predispose critically ill postoperative patients to CMV reactivation. Early CMV testing and timely antiviral therapy may facilitate diagnosis and help prevent clinical deterioration.

## Abbreviations

ATAAD
: acute type A aortic dissection
CMV
: cytomegalovirus
SMA
: superior mesenteric artery
TEVAR
: thoracic endovascular aortic repair

## INTRODUCTION

CMV enteritis is commonly reported in patients with overt immunosuppression, such as those receiving chemotherapy, transplant recipients, or individuals with advanced human immunodeficiency virus infection.^1)^ However, CMV enteritis can also occur in immunocompetent individuals exposed to severe physiological stress, particularly when accompanied by malnutrition or other immune-modulating factors.^2,3)^ The postoperative course following highly invasive procedures, such as surgery for ATAAD, may be associated with impaired immune function, thereby predisposing patients to opportunistic infections, including CMV enteritis. In cases of ATAAD complicated by malperfusion, gastrointestinal ischemia and subsequent nutritional deterioration may further increase this risk. Here, we report a case of CMV enteritis that developed during the postoperative course following extensive aortic surgery and concomitant thymectomy in a patient without prior immunosuppressive therapy.

## CASE PRESENTATION

A 65-year-old man was transferred to our hospital for surgical treatment of ATAAD. His medical history was notable only for hypertension, and he was not receiving corticosteroids or other immunosuppressive agents. Transthoracic echocardiography demonstrated preserved left ventricular function with moderate-to-severe aortic regurgitation. Contrast-enhanced CT revealed a dissection extending from the ascending to the abdominal aorta, with severe true lumen narrowing of the SMA and the brachiocephalic artery (**Fig. 1A**, **1B**). An anterior mediastinal mass suggestive of thymoma was also identified (**Fig. 1C**). The patient underwent emergency surgery comprising total arch replacement with a 28-mm J Graft (Japan Lifeline, Tokyo, Japan), implantation of a 31 × 150 mm Frozenix J-graft (Japan Lifeline), aortic valve replacement (Epic 27 mm; St. Jude Medical, Saint Paul, MN, USA), and concomitant thymectomy. Histopathological examination of the resected anterior mediastinal mass confirmed a type AB thymoma (**Fig. 2**). Cardiopulmonary bypass time was 424 minutes, the total operative time was 10 hours and 32 minutes, and the duration of circulatory arrest was 47 minutes. The minimum core temperature during circulatory arrest was 25°C. No direct intraoperative assessment of intestinal perfusion was performed during the initial emergency operation. Systemic hypoperfusion was monitored during and after surgery using serial arterial blood gas analyses. The blood lactate concentration peaked at 46 mg/dL (5.1 mmol/L) on POD 2 and subsequently decreased. Nevertheless, postoperative melena and persistent ileus raised concern for residual intestinal ischemia. Contrast-enhanced abdominal CT performed on POD 4 revealed persistent SMA narrowing and intestinal edema. On the same day, he underwent TEVAR using a 32–28–200 mm Relay Pro stent graft (Terumo, Tokyo, Japan), along with SMA stenting using a SMART stent (8 × 60 mm; Cordis, Fremont, CA, USA). After TEVAR, melena resolved immediately; however, he continued to have several episodes of watery diarrhea daily. After extubation on POD 6, persistent nausea and vomiting prevented him from tolerating oral intake. His nutritional status progressively deteriorated, with the serum albumin level decreasing from 4.5 g/dL preoperatively to 1.7 g/dL around POD 20. Clostridioides difficile toxin testing and routine stool cultures performed on POD 14 were negative. Because the gastrointestinal symptoms, low-grade fever, and elevated inflammatory markers persisted without an identified infectious source, CMV testing was performed on POD 20. Immunological evaluation on POD 21 showed serum IgG, IgA, and IgM levels of 907, 210, and 23 mg/dL, respectively. Flow cytometry demonstrated a CD19-positive B-cell proportion of 4.9%, a CD4-positive T-cell proportion of 58.2%, and a CD4/CD8 ratio of 2.58. The results obtained on POD 23 showed positive CMV antigenemia (C7-HRP: 42/50000 WBC), a blood CMV DNA level of 1.2 × 10^4^ IU/mL, positive CMV IgG (≥250.0), and negative CMV IgM (<0.85), consistent with CMV reactivation. Abdominal CT showed diffuse circumferential small-bowel wall thickening with submucosal edema (**Fig. 3**). Given the patient’s clinical condition, endoscopic biopsy was deferred. Based on the clinical course and virological and imaging findings, CMV enteritis was presumptively diagnosed. Intravenous ganciclovir (300 mg every 12 hours) was initiated on POD 23 and continued for 22 days. The CMV DNA level decreased to 2.8 × 10^2^ IU/mL on POD 30, CMV antigenemia became negative on POD 33, and blood CMV DNA became undetectable (<3.5 × 10^1^ IU/mL) on POD 41. Gastrointestinal symptoms and radiological findings also gradually improved. The patient was transferred to another facility on POD 90, although improvement in nutritional status and rehabilitation required a prolonged period.

**Fig. 1:**
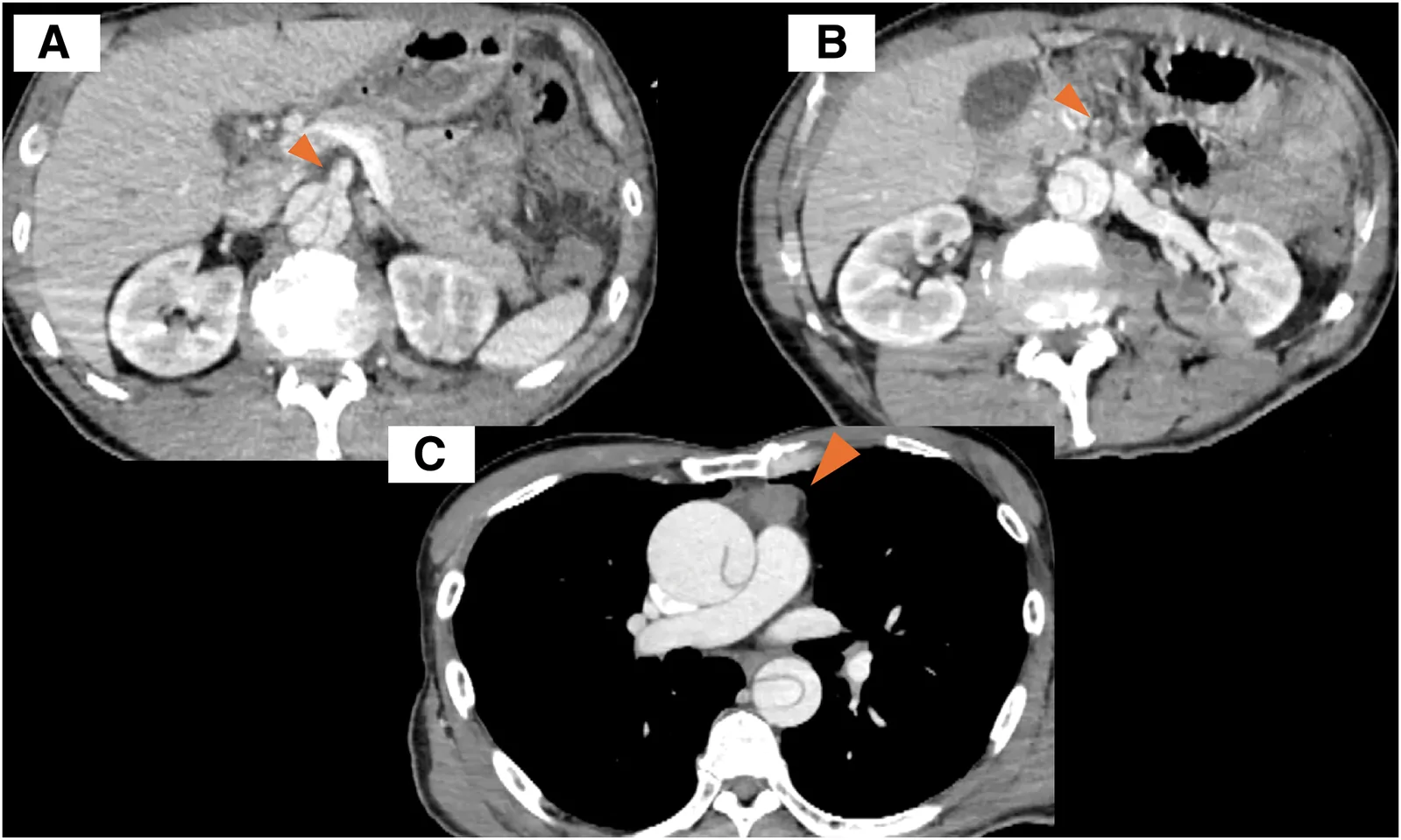
Preoperative contrast-enhanced CT demonstrated (**A**, **B**) narrowing of the true lumen at the origin of the SMA with distal occlusion (arrows), and (**C**) a 3.5 × 4.0 cm anterior mediastinal mass suggestive of thymoma (arrow). SMA, superior mesenteric artery

**Fig. 2:**
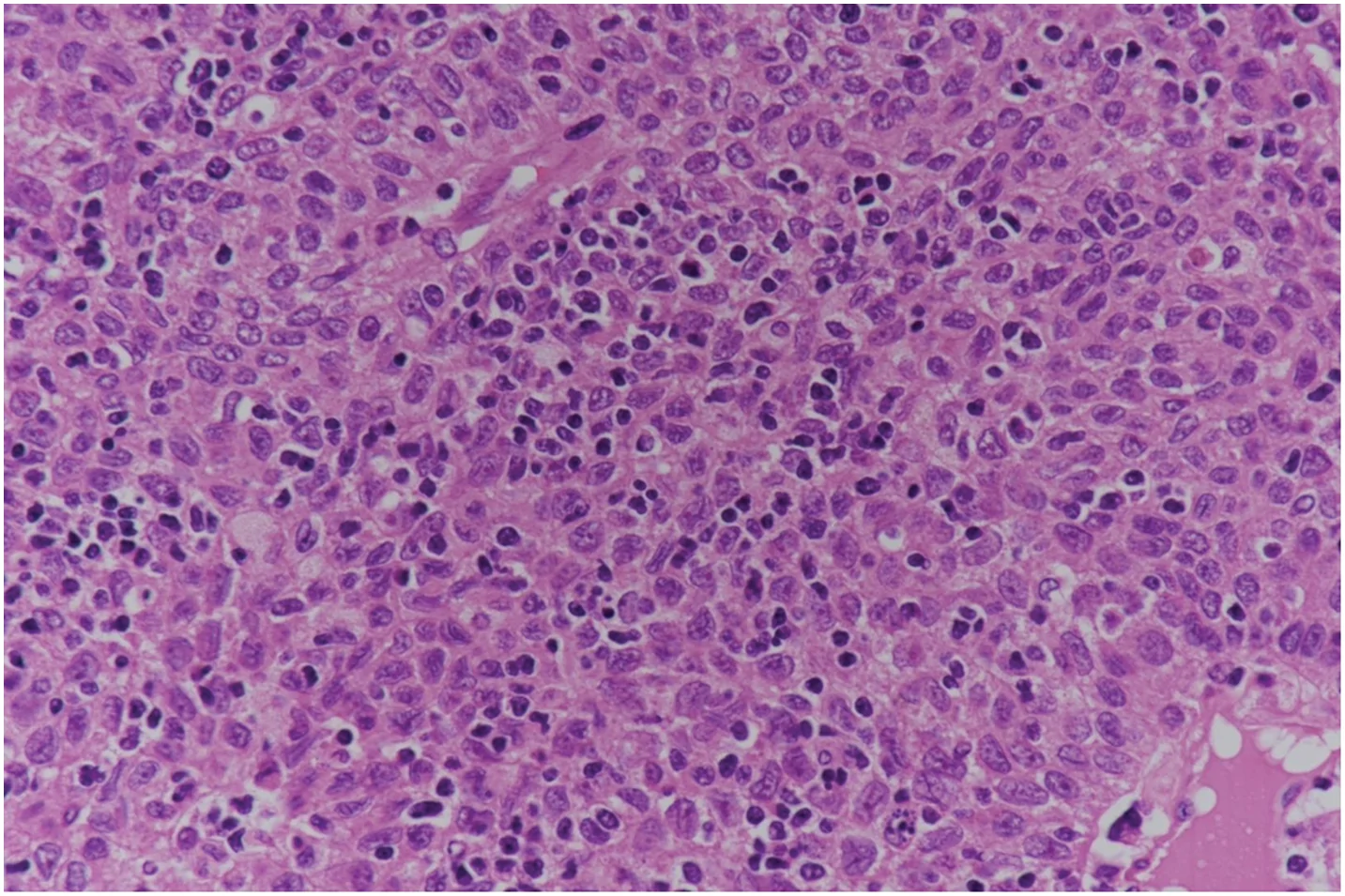
Histopathological examination of the resected anterior mediastinal mass revealed a type AB thymoma composed of a mixture of thymic epithelial cells and lymphocytes.

**Fig. 3:**
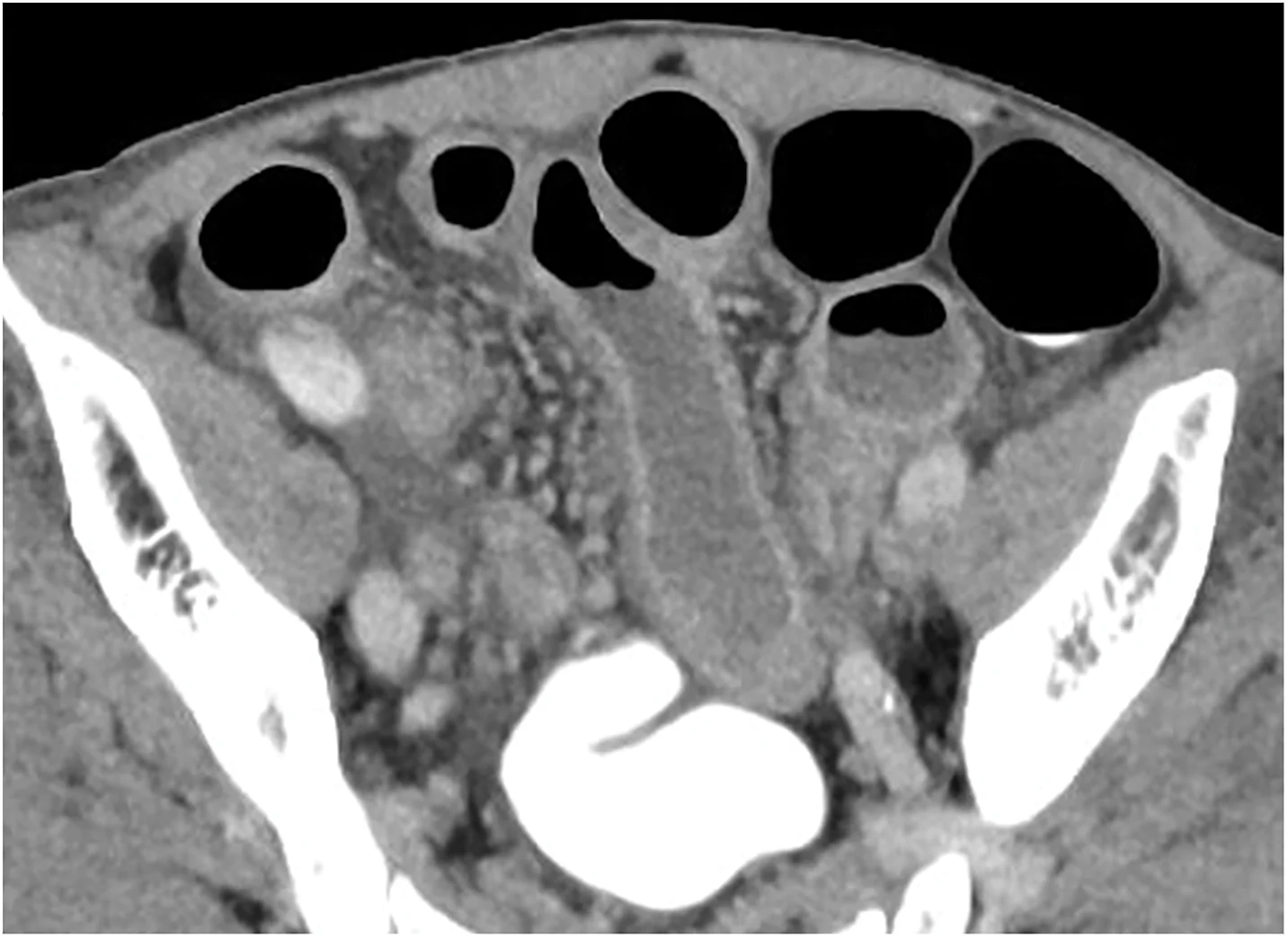
Postoperative CT demonstrated circumferential small bowel wall thickening with associated edema.

## DISCUSSION

Several factors may contribute to the development of CMV enteritis after ATAAD repair complicated by malperfusion. First, visceral malperfusion can cause bowel ischemia, leading to mucosal injury and disruption of the intestinal barrier. This ischemic damage may allow CMV to reactivate and invade locally, as reported in ischemic colitis and bowel obstruction even among immunocompetent patients.^4,5)^ Second, in the present case, the cardiopulmonary bypass time was 424 minutes, the total operative time was 10 hours and 32 minutes, and circulatory arrest lasted 47 minutes, all of which may have contributed to transient postoperative immune dysfunction through systemic inflammatory responses and transfusion-related immunomodulation.^6)^ Third, prolonged ileus and delayed nutritional recovery may further impair host defenses. In the present case, persistent poor oral intake was accompanied by a marked decrease in serum albumin levels from 4.5 g/dL preoperatively to 1.7 g/dL around POD 20, supporting the presence of substantial postoperative nutritional deterioration. Malnutrition can compromise T-cell-mediated immunity, phagocytic function, and mucosal immune responses that are critical for controlling latent viral infections.^7)^

Finally, the anterior mediastinal mass was histopathologically diagnosed as a type AB thymoma. Opportunistic infections, including CMV gastrointestinal disease, have been reported as initial manifestations of thymoma-associated immune dysregulation.^8)^ However, immunological evaluation on POD 21 revealed a low serum IgM level and a reduced proportion of CD19-positive B cells but no profound humoral or cellular immunodeficiency suggestive of Good’s syndrome. Because preoperative data were unavailable and the thymoma had been completely resected during the initial operation, neither the origin of these abnormalities nor a persistent thymoma-related effect could be established. Thus, the contribution of thymoma-associated immune dysregulation to CMV reactivation remains speculative.

The diagnosis of CMV enteritis was challenging in this case. Histopathological confirmation generally requires endoscopic biopsy showing CMV inclusion bodies or positive immunohistochemical staining. However, endoscopic biopsy may be impractical or high-risk in clinically unstable postoperative patients.^9)^ Given the patient’s clinical condition, biopsy was deferred. Clostridioides difficile toxin testing and routine stool cultures performed on POD 14 were negative, but specific testing for opportunistic fungal pathogens was not performed, and drug-induced enteritis could not be definitively excluded. After TEVAR, frequent watery diarrhea, nausea, and vomiting persisted. These symptoms may have been attributable to persistent post-ischemic ileus. Furthermore, the nonspecific CT findings of diffuse small-bowel wall thickening and submucosal edema could not be distinguished from residual ischemic changes after SMA malperfusion, complicating the diagnosis. CMV antigenemia and DNAemia, followed by decreased CMV DNA levels, clearance of antigenemia, and improvement in gastrointestinal symptoms and radiological findings after ganciclovir therapy, supported the presumptive diagnosis of CMV enteritis but did not confirm tissue-invasive disease. CMV enteritis was therefore considered the most likely diagnosis based on the overall clinical course.

Given these diagnostic limitations, CMV enteritis should be considered in postoperative patients with persistent unexplained fever and elevated inflammatory markers accompanied by prolonged gastrointestinal symptoms, including diarrhea, nausea, vomiting, or gastrointestinal bleeding. Particular attention is warranted in critically ill patients with intestinal ischemic injury, progressive nutritional deterioration, or a prolonged postoperative course, even in the absence of conventional immunosuppression. Early testing for CMV antigenemia and blood CMV DNA may facilitate timely diagnostic evaluation and treatment. From a perioperative perspective, prompt assessment and restoration of intestinal perfusion when indicated, close nutritional monitoring, and timely nutritional support may help preserve host defenses. Although these measures may not prevent CMV reactivation, early identification of high-risk patients and timely virological evaluation may reduce diagnostic delay and subsequent clinical deterioration.

## CONCLUSIONS

In conclusion, CMV enteritis should be considered in the differential diagnosis of unexplained fever accompanied by persistent gastrointestinal symptoms after open-heart surgery, particularly after operation of ATAAD complicated by malperfusion-related intestinal ischemia. Early CMV testing and timely antiviral therapy may prevent clinical deterioration and improve outcomes in this uncommon but serious condition.

## DECLARATIONS

### Availability of data and materials

The datasets supporting the conclusions of this article are included within the article.
